# Quality Indicators and Possible Ecological Risks of Heavy Metals in the Sediments of three Semi-closed East Mediterranean Gulfs

**DOI:** 10.3390/toxics7020030

**Published:** 2019-05-29

**Authors:** Nikolaos Stamatis, Nikolaos Kamidis, Pelagia Pigada, Georgios Sylaios, Emmanouil Koutrakis

**Affiliations:** 1 Hellenic Agricultural Organisation—Demeter, Fisheries Research Institute (F.R.I.), 64007 N. Peramos, 64007 Kavala, Greece; nikkami@inale.gr (N.K.); pigada@inale.gr (P.P.); manosk@inale.gr (E.K.); 2 Laboratory of Ecological Engineering and Technology, Department of Environmental Engineering, School of Engineering, Democritus University of Thrace, 67100 Xanthi, Greece; gsylaios@env.duth.gr

**Keywords:** heavy metals pollution, possible eco-risks, sediments, source identification, North Aegean Sea, Greece

## Abstract

Pollution with copper (Cu), lead (Pb), zinc (Zn), chromium (Cr), and nickel (Ni) heavy metals of the surface sediments collected from three semi-closed East Mediterranean Gulfs, namely Kavala, Strymonikos, and Ierissos Gulfs, North Aegean Sea, Greece, was investigated to evaluate potential benthic ecological risks. The mean concentrations of the studied metals decrease according to the order: Zn > Pb > Cr > Ni > Cu (176.50, 166.23, 127.41, 43.12, and 33.64 mg kg^−1^ dry weight). Quality indicators and possible ecological risks for metals in surface sediments were evaluated at 60 sampling sites of these three gulfs using the contamination factor (CF), the contamination degree (CD), the pollution load index (PLI), the geoaccumulation index (I*_geo_*), the potential risk factor (PRF*_i_*), and the potential ecological risk index (PERI). Based on I*_geo_*, the Ierissos Gulf sampling sites IER 2, 3, 7, and 9 exhibit moderate Pb pollution, whereas the sampling sites IER 6 and 8 show moderate to strong and strong Pb pollution, respectively. Based on the PRF*i* and PERI, the studied heavy metals did not pose any significant environmental risks for most of the investigated sites except IER 6 and 8 sampling sites, which may pose considerable environmental risk for Pb. To evaluate potential sources for each metal, multivariate techniques including hierarchical cluster analysis and ANOVA were used.

## 1. Introduction

Benthic habitats such as coastal zone sediments play an important role in the marine environment. Surface sediment contamination is one of the most important quality indicators for the assessment of potential ecological risks in coastal marine ecosystems. Therefore, the sediment may be regarded as a repository of pollutants, since after their entry in the water column, adsorption to finer particles and complexation may lead to their accumulation through the settling process into sediment [1,2,3,4,5]. Moreover, heavy metals may be biologically up taken by marine organisms, reaching the bottom sediments after their death [6,7]. Copper, Zn, Cr, and Ni are biologically essential elements since they have significant functions in the growth and health of biological systems [7], but they become toxic at higher concentrations [8]. Lead, as a non-essential metal, acts usually as a potent toxic species, even at very low concentrations. Its bioaccumulation in tissues [8] leads to intoxication, dysfunction of a variety of organs, cellular and tissue damage, decreased fertility, and cell death [9,10,11,12,13,14,15].

Some common anthropogenic activities that contribute to the release of Cu, Pb, Zn, Cr, and Ni species into the coastal marine environment consist in the following: mining operations, effluent discharges from fertilizer factories, oil refineries, and municipal sewage, spills during shipping and terminal transfers, intense agricultural activities, leaks of oil from offshore platforms, and usage of anti-corrosive and anti-fouling paints. Furthermore, river discharges, containing both natural and anthropogenic Cu, Pb, Zn, Cr, and Ni species contribute to the increase in metal contents of the coastal environment [16,17,18,19]. 

**Figure 1:**
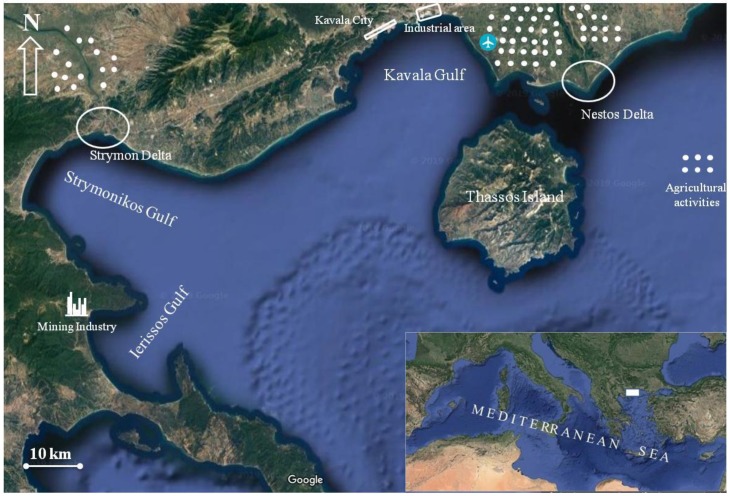
Map of main terrestrial activities in the watersheds of the three gulfs at northern Aegean Sea: Kavala Gulf; Strymonikos Gulf and Ierissos Gulf.

The Gulf of Kavala is a shallow marine ecosystem in the North Aegean Sea, flanked by the Symvolon Mountains to the west, the island of Thassos to the southeast, and the Nestos delta plain to the east and north (Figure 1). The gulf is approximately 25 km long from west to east and 12 km wide from north to south, with maximum water depths of about 45 m. Coastal industries including fertilizer and crude oil treatment plants for desulfurization and natural gas liquids extraction facilities, offshore oil platforms, pipelines, docks where ships routinely discharge oily ballast waters, intense touristic (western and central parts of the gulf area), and agricultural activities (eastern part of the gulf—Chrysoupolis plain), as well as municipal effluents (wastewater treatment plants of Kavala city and Palio settlement), are the major anthropogenic sources, supplying important loads of the above metals to the gulf’s environment. Moreover, Nestos River supplies most of the fine-grained sediments of the Kavala’s Gulf area [20]. 

The Strymon River originates from the Vitosha Mountain in Bulgaria, flows through the central part of the metamorphic basement of the Rhodope Mountains, passes through the dam lake Kerkini, and outflows into Strymonikos Gulf in the North Aegean Sea (Figure 1). Its drainage basin is rich in Cr and Ni maphic and ultramaphic rocks (ophiolites), and thus weathering is expected to lead to elevated sediment contents in such metals [21]. The impact of Strymon River mineralogy on the geochemistry of the Strymonikos Gulf sediments has been well investigated in previous studies [22,23]. Furthermore, Strymon River water carries agricultural wastes originating from activities in the plain of Serres valley, as well as industrial effluents and domestic sewage from municipalities located along its banks, in Greece and Bulgaria [24]. 

The Gulf of Ierissos, located to the south of Strymonikos Gulf, has an elliptical shape and NW-SE orientation with a 10 km opening in length towards the Strymonikos Gulf (Figure 1). In its restricted basin, different types of mineralization viewed as porphyritic or skarn Cu and Pb-Zn-Au-Ag carbonate-replacement were located [25]. The hydrographic network of the gulf is limited, with some torrents and streams outflowing seasonally at local level. The Cassandra mines exploiting the mixed sulfide ores of Chalkidiki are the most important source of the gulf’s environmental degradation. The mining activity and the processing of ores went on from as early as the 7th century BC. In the last few decades, mining included dispersal of mine tailings into the nearshore coastal area in the vicinity of Stratoni. Trawling is not allowed in the Ierissos Gulf, although it is a commercial fishing ground.

The water and the sediments in the above three gulfs contaminated in toxic pollution including the studied heavy metals, could have adverse effects to phytoplankton and other organisms or benthic communities, most of which form the basis for the marine food chain. Thus, the objective of the present study is (1) to assess the associated possible ecological risks of the existent concentration of five (Cu, Pb, Zn, Cr, and Ni) heavy metals in the surface sediments of the three neighboring gulfs by analyzing data from 60 sampling sites, (2) to assess possible ecological risks associated to sediment contamination in heavy metals using various indexes, such as CF, CD, PR, PLI, I*_geo_*, PRF*_i_*, and PERI, and (3) to identify potential sources of these metals, using statistical methods. This research approach could be helpful to understand the sediment quality distribution under the effects of the existed anthropogenic and/or natural activities, and thus support the strategic management of pollution with heavy metals in this specific coastal area.

## 2. Materials and Methods 

### 2.1. Sampling Methodology

**Figure 2:**
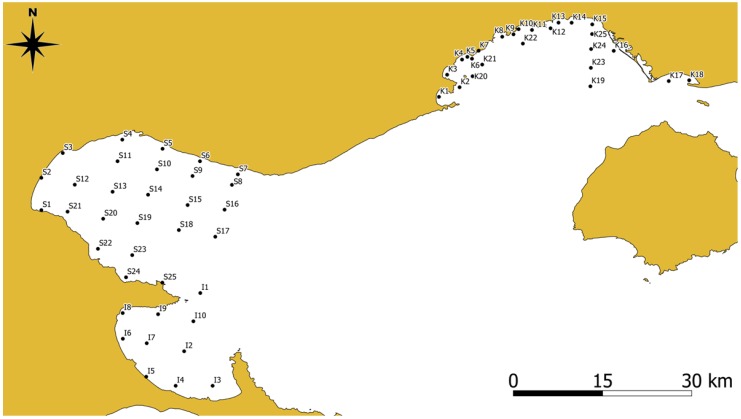
Map of the 60 sampling sites at the three gulfs at North Aegean Sea: Kavala Gulf (KAV 1–25); Strymonikos Gulf (STR 1–25) and Ierissos Gulf (IER 1–10).

The sampling strategy adopted aimed at detecting pollution sources as local point and non-point pollution sources, such as effluent discharges from mining operations or fertilizer factories, oil desulfurization plant and municipal sewage outflows, intense agricultural activities, possible spills during shipping transfers, leaks of oil from offshore drilling platforms, and usage of anti-corrosive and anti-fowling paints from the local shipping repair yards, as shown in Figure 1 and Figure 2. Especially in the Strymonikos Gulf, sampling focused on detecting river’s Delta influences in the adjacent marine environment. The sampling sites extended over the nearshore and the offshore areas covering all potential trace metals sources in the three gulfs. The detailed information for the study area including climate, regional landform, and geology, the water and suspended particulate matter geochemistry of the three gulfs, and adjacent land use could be found in our previous research works or in research works from other researchers [16,17,18,19,20,21,22,23,24].

The sediment samples were carefully collected to avoid disturbance with a Van Veen stainless-steel grab (dimensions 40 × 40 cm) from the upper 5 cm, during four sampling cruises (240 samples in total, 60 per season). The upper 5 cm layer was collected because it is more chemically active than the deeper layers, and substance exchanges between sediment and water occur in this layer. After collection, sediment samples were immediately placed at 0 °C, on board. Subsequently, until metal analyses, the samples were stored at −28 °C in plastic vessels, cleansed with 1N HNO_3_ for 1 h and rinsed thoroughly with double distilled deionized water.

### 2.2. Heavy Metal Analyses and Quality Control

In the chemistry laboratory, sediment samples, after defrosting, were passed through a 1 mm plastic sieve, dried at 50 °C up to a constant weight, and homogenized in a porcelain mortar with a pestle. A sediment sub-sample of 0.25 g was digested in a mixture of 4 mL of aqua regia and 1 mL of concentrated HF for 30 min using a Q-6000 microwave digestion system (Questron, Mississauga, ΟΝ, Canada). The resultant solutions were then diluted to a 25 mL volume (Erlenmeyer flask) with double distilled deionized water. Quantitative determinations of Cu, Pb, Zn, Cr, and Ni species were accomplished in triplicate using an Analyst 800 atomic absorption spectrometer (Perkin Elmer, Überlingen, Baden-Württemberg, Germany) equipped with a transversely heated graphite furnace (THGA) and longitudinal Zeeman-effect background corrector, AS-800 autosampler, 8-lamp turret, PE AA accessory cooling system, and the WinLab32 PC work station. The sediment material was always handled using plastic materials that were washed with 1N HNO_3_ and rinsed with double distilled deionized water to avoid any metal contamination. Acid washed glassware, analytical grade reagents, and double distilled deionized water were used for the sediment analysis. The analyses were carried out in triplicate, and the significance level was set at 0.05. Detection limits (in μg kg^−1^) were 0.014, 0.05, 0.02, 0.004, and 0.07 for Cu, Pb, Zn, Cr, and Ni, respectively. Analytical quality control was achieved using certified reference material for marine sediment (BCSS-1, National Research Council of Canada). The recovery percentage of Cu, Pb, Zn, Cr, and Ni concentrations from the reference material was (%) 98.77, 98.33, 98.15, 97.61, and 96.32, respectively. In order to determine the precision of the analytical procedure, samples from 5 sediments were analyzed four times. The standard deviation for these samples was calculated to (%) 1.9, 2.5, 2.2, 2.7, and 3.1 for Cu, Pb, Zn, Cr, and Ni, respectively, reaching the conclusion that precision of analysis is at satisfactory levels. 

### 2.3. Calculation of Metal Enrichment in Sediment Samples

As described below, several different methods were used to express heavy metal pollution in the studied sediments. Due to the lack of relevant background data for the unpolluted sediment in the area under investigation, the background heavy metal concentrations (pre-industrial era contamination levels) from the closely located Alexandroupolis Gulf, Northeast Aegean Sea, provided by Kanelopoulos et al. [26] were used. The geochemical background values for the bottom sediments were as follows: 27.0 for Cu, 12.0 for Pb, 74.0 for Zn, 63.0 for Cr, and 43.0 mg kg^−1^ for Ni.

#### 2.3.1. The Contamination factor (CF) and Contamination Degree (CD) 

CF and CD are widely used proxies for the estimation of the anthropogenic impact on sediment chemistry, referring to the enrichment degree of metal concentrations in sediments investigated relative to uncontaminated background levels (baseline) [27,28,29,30]. While CF is calculated for individual elements, CD provides overall information regarding the sediment contamination of the study sites. CF and CD are expressed as:

CF = C*_m_*/C*_b_*

$$CD=\sum_{i=1}^{n}\left(CF\right)$$

where C*_m_* denotes the content of the specific heavy metal investigated, and C*_b_* is the local uncontaminated background level for the same metal. Generally, CF values are categorized at four classes, e.g. CF < 1; 1 ≤ CF < 3; 3 ≤ CF < 6 and CF ≥ 6, indicating low, moderate, considerable, and very high contamination, respectively [27]. Likewise, CD values are categorized at four classes as well, e.g. CD < 6; 6 ≤ CD < 12; 12 ≤ CD < 24 and CD ≥ 24 indicating low, moderate, considerable, and very high contamination, respectively [27,31].

#### 2.3.2. The Pollution Load Index (PLI)

The overall contamination sediments in heavy metals content of sediments at the studied sites was evaluated using the PLI, calculated with the following equation [32].

PLI = (CF_1_ × CF_2_ × ……× CF*_n_*)^1/*n*^

where *n* is the number of metals and CF the contamination factor of metals studied. PLI values close to 0 or 1 indicate perfection or baseline levels of pollutants present, respectively; on the contrary, PLI values >1 indicate progressive deterioration of the area [33]. 

#### 2.3.3. The Geoaccumulation Index (I*_geo_*)

To understand the current status of the environment and the heavy metal contamination in respect to the natural environment for the Kavala Gulf, Strymonikos Gulf, and the Ierissos Gulf, the geoaccumulation index (I*_geo_*) was applied using the following equation [34,35]:

I*_geo_* = log_2_[C*_n_*/(1.5 × C*_bn_*)]

where C*_n_* is the concentration of the examined metal in the studied surface sediment sample, C_*bn*_ is the geochemical background concentration of a given metal [26,36], and the factor 1.5 is the matrix correction factor of the background concentration due to the lithogenic effects. Müller [37] has distinguished seven classes of I*_geo_* (Table 1). 

### 2.4. Ecological Risk Assessment

A potential ecological risk index (PERI) was used to evaluate the potential ecological risk of heavy metals in the sediments [27,31]. The risk index was calculated using the following equations:

PRF*_i_* = TR*_i_* × C*_mi_*/C*_bi_*

$$\mathrm{PERI}=\sum_{i=1}^{5}\left({\mathrm{PRF}}_{i}\right)$$

where PRF*_i_* is the potential risk factor of a given pollutant; TR_i_, the toxic response of metals (Cu = Pb = 5; Zn = 1; Cr = Ni = 2) [38]; C*_mi_*, the concentration of metal i in the sediment; and C*_bi_*, the regional background of the metal i in sediments. The following thresholds were used to interpret the PERI and PRF*_i_* values [39]:

Low risk:PRFi < 30; PERI < 100Moderate risk:30 < PRFi < 50; 100 < PERI < 150Considerable risk:50 < PRFi < 100; 150 < PERI < 200Very high risk:100 < PRF_i_ < 150; 200 < PERI < 300Disastrous risk:PRF_i_ > 150; PERI > 300

### 2.5. Statistics

For the interpretation of the data of the chemical analysis the following methodology was used: (1) descriptive statistics (mean, range, standard deviation) and correlations between elements and sampling areas (gulfs) were performed using the Stat soft Statistica 10 software (Statsoft Inc., Tulsa, OK, USA.). The results obtained were expressed as the mean ± standard deviation (SD) and were analyzed by means of analysis of variance (ANOVA). A value of *p* < 0.05 was considered to indicate statistical significance; (2) cluster analysis among sampling sites and among metals were applied to the standardized through *z*-scale - transformed data [40] in order to eliminate the different unit scales between metals [41]. For the hierarchical cluster analysis the Ward’s method was chosen and the Squared Euclidean distances was applied as distance/similarity measure, so all objects/results should be characterized by internal homogeneity and external heterogeneity [42]; (3) Pollution ratio for each metal and for its subarea was calculated; Pollution ratio is the average metal concentration of a region divided by the metal concentration of the subarea I, which is the area with the lowest heavy metal content; (4) inter-element Pearson correlation coefficients (*r*) were also calculated. 

## 3. Results

### 3.1. Descriptive Statistics

Summary statistics for metal concentrations in the surface sediments of each gulf of the studied area and each metal are presented in Table 2.

Mean values of Cu, Pb, and Zn in Kavala and Strymonikos Gulf show no significant differences between samples (ANOVA, *p* < 0.05), whereas those of all metals exhibited higher mean concentrations in Ierissos Gulf compared to Kavala and Strymonikos Gulf samples. Mean values of Cr and Ni were also significantly higher in Strymonikos compared to Kavala Gulf samples.

### 3.2. Cluster Analysis 

**Figure 3:**
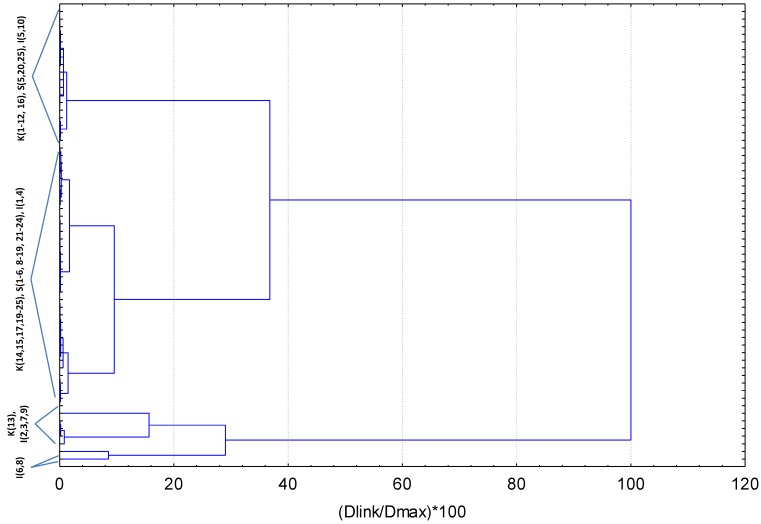
Dendrogram for hierarchical cluster analysis of five metals concentrations in sediments collected from 60 sites in three neighboring gulfs of North Aegean Sea, Greece. Dlink/Dmax represents the quotient of the linkage for a particular sampling site divided by the maximum distance; the quotient is multiplied by 100 for standardizing the linkage distance on the *y*-axis.

The dendrogram produced by the cluster analysis for all sampling sites is presented in Figure 3. The dendogram explained the similarity between a single object and the entire dataset and presently identified four principal groups, corresponding to the subareas I, II, III, and IV. 

The sampling sites included in the various subareas of the study region as well as mean, range concentration values, and pollution ratios per subarea are given in Table 3. Mean metal subareal contents can be ranked by abundance in the marine sediments of the study area as follows: sub I < sub II < sub III < sub IV. 

Subarea I is characterized by the lowest means for all metals and could be used as a reference. It contains the western stations of Kavala Gulf affected from touristic (KAV 1–7) and some urban activities (KAV 8–12), as well as a single station positioned at the north of the gulf (close to oil desulfurization plant). This subarea also includes three inshore Strymonikos Gulf stations and two stations from Ierissos Gulf: IER 5 located close to the shore and IER 10 positioned at the gulf’s entrance. Subarea II showed intermediate mean metal concentrations and includes all but one (KAV 13), the remaining eastern (KAV 16–18) and the offshore Kavala Gulf sites (KAV 19, KAV 20–25), the rest of Strymonikos sites regardless of their position (nearshore, offshore, near river plume, etc.), showing gulf’s homogeneity, and two Ierissos Gulf sites, the southernmost IER 4 and IER 1-site located at the boundary between Strymonikos and Ierissos Gulfs. Subarea III demonstrates elevated mean values for all metals, containing five sites in total: KAV 13 located at the industrial part of Kavala Gulf (close to phosphoric fertilizer plant) and four Ierissos sites (IER 2, 3, 7, and 9) positioned at the gulf’s intermitter-depth transect. Finally, subarea IV includes the most polluted sites of Ierissos Gulf, mostly influenced by mining activities. 

**Figure 4:**
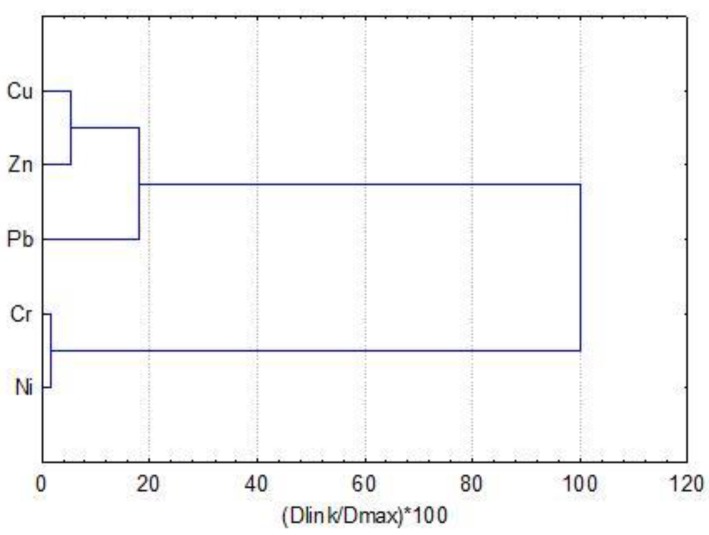
Dendrogram for hierarchical clusters analysis of five metals concentrations in sediments collected from three neighboring gulfs in North Aegean Sea, Greece. Dlink/Dmax represents the quotient of the linkage for a metal divided by the maximum distance; the quotient is multiplied by 100 for standardizing the linkage distance on the *y*-axis.

The cluster analysis applied for the five investigated metals revealed two individual groups (Figure 4): the first consisted by Cr and Ni, indicating their very strong inter-relation (Pearson *r* = 0.98, *p* < 0.05) and the second by Cu, Pb, and Zn (Pearson *r* = 0.80–0.94, *p* < 0.05). 

### 3.3. Metal Enrichment Factors in Sediment Samples, Contamination Factor (CF), Contamination Degree (CD), Pollution Load Index (PLI), and Geoaccumulation Index (I_geo_)

According to mean and range CF values (Table 4), all the sites of three gulfs indicated low to moderate contamination for Cu, considerable to very high contamination for Pb, moderate contamination for Zn (except IER 6 and IER 8 sites, which showed very high contamination for Zn), low to moderate contamination for Cr, (except STR 6 and IER 5 sites with considerable contamination), and finally low to moderate contamination for Ni (except IER 6 site, which showed considerable contamination for Ni). Very high contamination degrees (CD > 24) 49.84, 57.84, 27.50, 157.63, 74.48, 209.74, 54.89, and 38.03 for the IER 2, 3, 4, 6, 7, 8, 9, and KAV 13 sampling sites respectively, indicated the degree of serious anthropogenic pollution, mainly in Ierissos Gulf and secondarily in Kavala Gulf. All other sites of the study areas showed low contamination degree (Table 4). Mean CD values of the studied areas can be ranked as follows: Strymonikos Gulf < Kavala Gulf < Ierissos Gulf. 

The highest metal pollution load indexes (PLI) were recorded for IER 6 and IER 8 sites (11.50 and 8.17, respectively) followed by IER 7 (6.68), IER 3 (5.65), IER 9 (5.22), IER 2 (4.98) and KAV 13 (3.41); while the lowest PLI was recorded for KAV 7 site (0.20). According to mean PLI values, the pollution in the study area follows a descending order of: Ierissos Gulf > Strymonikos Gulf > Kavala Gulf (Table 4). In general, results of PLI are in agreement with these shown from CD.

The I*_geo_* values for the three studied gulf areas were represented in Table S1. According to the Müller’s scale, the calculated I*_geo_* values for Cu, Pb, Zn, Cr, and Ni in all studied sediments from Kavala and Strymonikos Gulfs belong to class zero, indicating that sediments in all stations are unpolluted by these metals. On the other hand, sediments of Ierissos Gulf sites fluctuated from unpolluted (IER 1, 4, 5, and 10) to moderately (IER 2, 3, 7, and 9 with corresponding I*_geo_* values of 1.03, 1.24, 1.66, and 1.19), moderately/strongly (IER 6; I*_geo_* = 2.85), and strongly polluted (IER 8; I*_geo_* = 3.37) by Pb (Table S1). 

### 3.4. Potential risk factor and ecological risk index (PRF_i_ and PERI)

Table S2 summarizes the calculated values of PRF_i_ and PERI for metals in the sediments of the three studied gulf areas. Among the studied heavy metals, Pb reported the highest ecological risk, because of its considerable high toxicity factor (PRF_i_) in some sampling sites of Ierissos Gulf. Moreover, the PRF_i_ Pb mean value is calculated by 5.77 ± 12.24, which indicated that Pb content varied greatly through the sampling sites of the three gulfs. About 97% of the sampling sites recorded PRF_i_ Pb values lower than 30 indicating a low risk from Pb, while 3% of the sites recorded PRF_i_ Pb higher than 50, which indicates a considerable risk. All sampling sites in the studied three gulfs recorded PRF_i_ << 30 for the other four metals Cu, Zn, Cr, and Ni (Table S2), which is a considerable low risk where their corresponding averages were (0.23 ± 0.26, 0.03 ± 0.04, 0.06 ± 0.04, and 0.05 ± 0.03, respectively). Overall, the ecological risk of heavy metals in the surface sediments for the study three gulfs area recorded a low risk (PERI << 100). The obtained data indicated that the sediments in the three studied gulfs area have the following order: Kavala Gulf (2.10 ± 1.48) < Strymonikos Gulf (3.51 ± 1.31) < Ierissos Gulf (22.87 ± 25.35).

## 4. Discussion

The results of the various methods for calculating heavy metal enrichment in sediments and possible environmental risks from three neighboring gulfs in North Aegean Sea demonstrate that sediments of two sites in Ierissos Gulf (IER 6 and 8) located in the vicinity of the ore unloading terminal of the mining operations (‘flotation’) in Ierissos Gulf (Stratoni Bay) and sediments of the site KAV 13, adjacent to phosphoric fertilizer plant in Kavala Gulf exhibit the highest contamination in the examined metals.(Figure 2). Regarding Ierissos Gulf, the concentrations of Pb, Cu, and Zn were found lower than those reported by Pappa et al. [43], probably due to the station allocation inside the gulf. In the present study an effort to cover the entire gulf was made, while Pappa et al. [43] examined the metal concentrations close to potential sources, giving additional emphasis to Stratoni harbor, the load out pier area that serves the mining operation and Ierissos commercial port to the south. The negative impacts of heavy metal elevated concentrations, which originate from mining and ore processing activities are well known from previous studies [44,45,46]. Also, sediments collected from most of the other sampling sites in Ierissos Gulf (directly affected from the mining activities) and from the central (urban activities) and deeper (oil offshore plant) parts of the Kavala Gulf showed increased CD, PLI, and PERI values. The degradation of the aquatic environment at the urban and industrial area of Kavala Gulf is also mentioned by other studies [17,47]. Point sources from municipal wastewater and the phosphoric fertilizer plant, as well as non-point sources from phosphogypsum leaching and agricultural activities, appear responsible for the local increase in the concentration of nutrients and lead concentrations [17,47]. On the contrary, sampling sites from the locations along the western (tourism and recreational activities) and eastern (aquaculture activities) coastline of Kavala Gulf, showed low values of the above metal pollution indexes. This area was used as “background area” in order to normalize Pb concentration and to assess Pb pollution inside Kavala Gulf [17].

Most of the sediments in Strymonikos Gulf show moderate to high and low values for the CD, PLI, and PERI indexes. Strymon River is the important anthropogenic source of metal pollution for the gulf as, it carries effluents from the industrial activities located along its banks, in Greece and Bulgaria, agricultural wastes originating from Serres valley, as well as domestic sewage. Moreover, drainage basin of the gulf contains maphic and ultramaphic rocks (ophiolites) rich in Cr and Ni, thus, the weathering products contain elevated concentrations of natural origin of such metals [21]. Furthermore, Richios River, which carries agricultural wastes from the Mygdonia (Lagadas) valley, and other streams flow into the gulf contributing to its environmental degradation. In general, it is well known that one of the main significant causes in the spatial distribution of pollutants in the coastal environment is the location of the pollutant sources and their direct discharge rates [48]. It occurs that comparison of the metal amounts from different sediments in the same or/and in different coastal areas showed that different stations have different amounts of metals due to the diversity in anthropogenic or natural polluting sources. In our study, the high Cu, Pb, and Zn contents in Ierissos and Kavala Gulf sediments can be attributed to the existence of industrial activities (mining-, fertilizer-, and oil production/purification-units) and the discharge of wastes from these factories to the adjacent coastal zone. On the other hand, the elevated Cr and Ni contents in the Strymonikos Gulf and their disproportionate high ranges compared to the high polluted areas (see Table 2 and Table 3) can be associated to the weathering products of ophiolites of the Strymon River. This is probably the main reason of Cr and Ni grouping as shown in Figure 4. Also, as shown by Sylaios et al. [24], Strymon River is capable to transfer pollutants in the entire gulf under high flow conditions.

Overall, the potential risk factor of heavy metals in the surface sediments for the studied gulfs recorded a low risk (PRF*_i_* << 30) except in IER 6 and IER 8 sampling sites PRF indicates a considerable risk, mostly due to Pb. However, the overall potential ecological risk (PERI) of the studied heavy metals in the surface sediments for the study area exhibited a low risk (PERI << 100). The highest number that PERI scored is at station IER 8. Further research is needed in terms of investigations of various mechanisms influencing metal impacts on organisms living in sediment or overlying water layers of the studied gulfs, like speciation, aging and weathering, limitations to uptake by organisms, as influenced by absorption and eliminator kinetics, etc. The I*_geo_* factor is not readily comparable to the other metal enrichment indexes in sediments due to the nature of the I*_geo_* calculation, which involves a background multiplication of 1.5 and a log function. Based on the classification system proposed for I*_geo_* indexes, Kavala Gulf and Strymonikos Gulf sediments are grouped on average as “unpolluted”. On the contrary, most of the sediments of Ierissos Gulf sites showed moderate to strong pollution in Pb. The I*_geo_* “unpolluted” designation is clearly inappropriate as part of an overall description of the heavy metal results for the sediments from our study area.

Although Cu, Zn, Cr, and Ni are biologically essential elements for the healthy development of the organisms in the coastal and other aquatic environments, they can become toxic to organisms’ life at levels higher than the threshold limits [49]. On the contrary, lead belongs to the non-essential elements and it is usually very toxic even at very low concentrations. Its bioaccumulation in marine benthic or other organism tissues is well documented [8,50,51]. In general, heavy metal contamination produces disturbances in the diversity, abundance, and biomass of marine benthic communities [52,53,54]. In the present study, the elevated metal content occurring in sediments of sites located near the industrial activities, especially in Ierissos Gulf, could cause adverse effects on the local benthic micro- and macro-fauna and flora, due to their bioaccumulation in tissues [50]. Due to the sediment quality degradation caused mainly by the mining activities during previous decades, the bottom trawl fishing throughout the entire Ierissos Gulf has been banned by law, since 1966 (Article 8 of the Greek Government Gazette No 248A). Trawlers’ main catch is benthic species living close to the sea bottom, which is the storage area of heavy metals. In addition, trawlers are able to produce mixing conditions at the bottom sediments, and as a result some metals may be transferred to the water column. On long-term basis, mining could increase sediment loads in the coastal drainage basin of Ierissos Gulf causing further release of toxic chemicals, some contained in exposed ore bodies and waste rock piles and some derived from ore-processing reactions. On short-term basis, mining distorted the surrounding benthic topography and removed of coastal benthic vegetation. The results of the present study illustrated that the large-scale industrial activities are provoking a huge effect on the quality of surface sediments in the studied gulfs of the North Aegean Sea.

## 5. Conclusions

The impact of anthropogenic and/or natural heavy metal pollution on three neighboring gulfs in the North Aegean Sea, Greece was evaluated using the contamination factor (CF), the contamination degree (CD), the pollution load index (PLI), the geoaccumulation index (I*_geo_*), the potential risk factor (PRF*_i_*), and the potential ecological risk index (PERI) per sampling site for Cu, Pb, Zn, Cr, and Ni in 60 surface sediment samples. 

IER 6 and IER 8 surface sediments from the Ierissos Gulf, as well as KAV 13 sediment from the Kavala Gulf showed the maximum values for CF, CD, PLI, PRF*_i,_* and PERI indexes, which means that the above sampling sites were the most severely polluted locations by the metals Cu, Pb, Zn, Cr, and Ni in both gulfs, respectively. Moreover, most of the other sediments in Ierissos Gulf (directly affected from mining) and in the central and deeper parts of Kavala Gulf (directly affected from industrial activities and oil offshore production) showed increased values of the above indexes. Based on the PRF*i* index, the studied heavy metals did not pose significant environmental risks for the most of the investigated sampling sites, except IER 6 and IER 8, which may pose considerable environmental risk for Pb.

The geoaccumulation indexes (I*_geo_*) are distinctly variable and suggest that sediments in the various Ierissos gulf samples range from unpolluted to moderately, moderately/strongly, and strongly polluted with respect to the Müller’s scale and to the analyzed Pb. Sediments from the other two gulfs as well as from Ierissos Gulf are unpolluted by all investigated metals and by Cu, Zn, Cr, and Ni, respectively. This finding, for uncontaminated sampling sites by using I*_geo_* index, did not correspond with the other indexes findings about metal pollution impact in the study area.

Results of Pearson correlation matrix and cluster analysis distinguished the different metal sources for all the examined areas and indicated that Cr and Ni in the surface sediments derived primarily from natural (lithogenic) source, while Cu, Zn, and Pb were largely contributed to by the anthropogenic sources, such as mining activities in Ierissos Gulf and other industrial (fertilizer factory, oil offshore production) and urban activities in Kavala Gulf. The pollution level of heavy metals at the surface layer of sediments in the three gulfs decreased in the order of Pb ~ Zn > Cr > Ni ~ Cu.

In terms of an ecological risk assessment in these three ecosystems, further research is needed, such as studying of various mechanisms which could influence metal impacts on local organisms living on sediment or overlying water layers, like speciation, aging and weathering, limitations to uptake by organisms, as influenced by absorption and eliminator kinetics, etc. 

## Figures and Tables

**Table 1:** Müller’s classes for the geoaccumulation index.

I*_geo_* Value	Class	Quality of Sediment
≤0	0	Unpolluted
0–1	1	Unpolluted/moderately polluted
1–2	2	Moderately polluted
2–3	3	Moderately/strongly polluted
3–4	4	Strongly polluted
4–5	5	Strongly/extremely polluted
≥6	6	Extremely polluted

**Table 2:** Range, mean concentrations and standard deviation of heavy metals observed at Kavala, Strymonikos and Ierissos Gulfs (n.d. = not detected; SD = Standard Deviation).

Heavy Metal		Kavala Gulf	Strymonikos Gulf	Ierissos Gulf
Cu	Range	0.78–154.02	5.32–51.24	n.d.–205.88
	Mean	25.14	27.69	69.81
	SD	29.67	12.80	68.86
Pb	Range	18.12–203.28	23.41–130.46	52.91–2233.09
	Mean	52.79	91.10	637.66
	SD	36.21	35.06	713.82
Zn	Range	48.15–1024.70	24.31–158.99	39.63–926.8
	Mean	139.78	110.67	420.08
	SD	187.09	38.68	316.66
Cr	Range	23.16–185.28	29.24–213.04	17.12–364.23
	Mean	80.79	148.72	190.71
	SD	47.09	55.01	105.64
Ni	Range	0.84–50.00	9.06–74.51	2.1–144.30
	Mean	22.32	53.62	68.88
	SD	16.89	19.89	45.88

**Table 3:** Sampling sites and levels of heavy metals in various subareas of the study region.

Area	Sampling Sites		Heavy Metal Concentrations (mg kg^−1^)				
			Cu	Pb	Zn	Cr	Ni
I	KAV(1-12,16) STR(7,20,25) IER(5,10)	Mean	11.02	42.61	72.90	42.39	9.46
		Range	0–44.06	18.12–83.54	24.31–135.61	17.12–72.89	0.84–0.46
II	KAV(14,15,17-25) STR(1-6,8-19,21-24) IER(1,4)	Mean	29.63	90.82	125.17	152.49	53.50
		Range	7.69–51.24	23.41–226.47	53.2–260.81	74.26–213.04	26.51–74.51
		PR	2.69	2.13	1.72	3.60	5.66
III	KAV(13) IER(2,3,7,9)	Mean	77.92	466.44	585.42	218.51	80.98
		Range	50.52–154.02	203.28–683.47	387.48–1024.7	79.51–269.14	11.78–112.70
		PR	7.07	10.95	8.03	5.15	8.56
IV	IER(6,8)	Mean	192.50	1895.58	908.11	268.87	88.17
		Range	179.11–205.88	1558.06–2233.09	889.42–926.80	173.51–364.23	32.03–144.3
		PR	17.47	44.49	12.46	6.34	9.32

PR = Pollution Ratio.

**Table 4:** The contamination factor (CF) ^1^, geoaccumulation index (I*_geo_*) ^2^ contamination degree (CD) ^3^, and the pollution load index (PLI) ^4^ of heavy metals in surface sediments from three gulfs, North Aegean Sea, Greece: Mean and Range values.

Heavy Metal	Index	Kavala Gulf		Strymonikos Gulf		Ierissos Gulf	
		Mean	Range	Mean	Range	Mean	Range
Cu	CF	0.93	0.03–0.93	1.03	0.20–1.90	1.65	0.00–7.63
	*I*_geo_	−6.21	−10.45–(−2.83)	−5.54	−7.68–(−4.42)	−4.35	−7.04–(+3.37)
Pb	CF	4.40	1.51–4.40	7.59	1.95–10.87	26.64	4.41–186.09
	*I*_geo_	−2.24	−3.58–(−0.09)	−1.40	−3.21–(−0.73)	0.67	−2.03–(3.37)
Zn	CF	1.89	0.65–1.89	1.50	0.33–2.15	3.20	0.54–12.52
	*I*_geo_	−6.26	−7.41–(−3.00)	−6.34	−8.40–(−5.69)	−4.88	−7.70–(−3.15)
Cr	CF	1.28	0.37–1.28	2.36	0.46–3.38	2.60	0.27–5.78
	*I*_geo_	−6.48	−8.01–(−5.01)	−5.47	−7.67–(−4.80)	−5.34	−8.44–(−4.03)
Ni	CF	0.52	0.02–0.52	1.25	0.21–1.73	1.37	0.05–3.36
	*I*_geo_	−7.61	−11.69–(−5.79)	−5.85	−8.26–(−5.22)	−5.95	−10.37–(−4.26)
	CD	9.02	13.72–66.03	2.74	4.01–5.89	38.03	19.49–209.74
	PLI	1.24	0.20–3.41	2.00	0.48–2.92	4.82	0.00–11.50

^1^ CF < 1, low contamination; 1 ≤ CF < 3, moderate; 3 ≤ CF < 6, considerable; 6 ≤ CF, very high [27,31]. ^2^ I*_geo_* < 0, unpolluted; 0 ≤ I*_geo_* < 1, unpolluted to moderately polluted; 1 ≤ I*_geo_* < 2, moderately polluted; 2 ≤ I*_geo_* < 3, moderately to strongly polluted; 3 ≤ I*_geo_* < 4, strongly polluted; 4 ≤ I*_geo_* < 5, strongly to extremely polluted; 5 ≤ I*_geo_*, extremely polluted [29,37]. ^3^ CD < 6, low contamination; 6 ≤ CD < 12, moderate; 12 ≤ CD < 24, considerable; 24 ≤ CD, very high [27]. ^4^ 0 ≤ PLI < 1, unpolluted or baseline levels of pollutants, 1 ≤ PLI metal pollution is present.

## Supplementary Materials

The following are available online at https://www.mdpi.com/2305-6304/7/2/30/s1, Table S1: Geoaccumulation index (I*_geo_*) values of heavy metals of sediment samples in the three investigated gulfs, North Aegean Sea, Greece, Table S2: Potential risk factor (PRF_i_) and potential ecological risk index (PERI) of heavy metals in sediment samples of three investigated gulfs, North Aegean Sea, Greece.
