# Supplementary material for: Quality Indicators and Possible Ecological Risks of Heavy Metals in the Sediments of three Semi-closed East Mediterranean Gulfs
Source: Toxics. 2019 May 29;7(2):30. doi: 10.3390/toxics7020030 (PMC6632002; doi:10.3390/toxics7020030)
Supplement: Supplementary file 1 [file toxics-07-00030-s001.pdf]

Toxics **2019**, *7*, 30; doi:10.3390/toxics7020030 www.mdpi.com/journal/toxics

Supplementary Meterials: Quality Indicators and Possible Ecological Risks of Heavy Metals in the Sediments of Three Semi-closed East Mediterranean Gulfs

Nikolaos Stamatis, Nikolaos Kamidis, Pelagia Pigada, Georgios Sylaios and Emmanouil Koutrakis

Table S1. Geoaccumulation index (I_{geo}) values of heavy metals of sediment samples in the three investigated gulfs, North Aegean Sea, Greece.

Sampling location	Site	Cu	Pb	Zn	Cr	Ni
Kavala Gulf	KAV1	−6.83	−2.65	−6.40	−7.50	−7.89
	KAV2	−9.28	−3.58	−7.20	−7.99	−10.80
	KAV3	−7.15	−2.65	−6.62	−7.41	−8.54
	KAV4	−8.18	−2.73	−7.11	−7.64	−8.78
	KAV5	−9.16	−3.03	−7.04	−8.00	−10.17
	KAV6	−7.68	−2.55	−6.77	−7.38	−8.48
	KAV7	−10.45	−3.10	−7.41	−8.01	−11.69
	KAV8	−5.34	−1.37	−5.92	−6.90	−8.01
	KAV9	−5.59	−1.46	−6.29	−7.33	−9.80
	KAV10	−6.62	−2.36	−6.83	−6.41	−7.63
	KAV11	−5.33	−2.48	−6.31	−6.35	−7.40
	KAV12	−4.63	−1.95	−6.14	−6.63	−8.05
	KAV13	−2.83	−0.09	−3.00	−6.23	−7.88
	KAV14	−4.79	−2.76	−6.32	−5.20	−5.99
	KAV15	−5.52	−2.76	−6.20	−5.65	−6.14
	KAV16	−7.39	−2.96	−7.14	−6.78	−7.77
	KAV17	−6.05	−2.43	−6.51	−5.99	−6.61
	KAV18	−4.87	−2.18	−5.93	−5.75	−6.16
	KAV19	−5.60	−1.90	−6.20	−5.86	−6.40
	KAV20	−5.30	−1.43	−5.79	−5.63	−6.04
	KAV21	−5.11	−1.72	−5.76	−5.55	−6.07
	KAV22	−5.25	−1.75	−5.88	−5.48	−5.99
	KAV23	−5.70	−2.21	−5.96	−5.72	−6.22
	KAV24	−5.35	−1.76	−5.89	−5.56	−6.01
	KAV25	−5.23	−2.05	−5.93	−5.01	−5.79
Strymonikos Gulf	STR1	−6.08	−2.46	−6.51	−5.45	−5.75
	STR2	−6.71	−3.21	−7.27	−5.85	−6.30
	STR3	−5.22	−2.27	−6.44	−5.06	−5.44
	STR4	−4.70	−1.54	−5.92	−4.99	−5.27
	STR5	−4.98	−1.45	−6.11	−5.31	−5.68
	STR6	−4.93	−1.22	−6.01	−5.02	−5.32
	STR7	−7.29	−2.86	−7.57	−6.80	−7.08
	STR8	−5.27	−1.39	−6.25	−5.33	−5.60
	STR9	−4.96	−1.24	−6.02	−5.06	−5.48
	STR10	−4.91	−1.02	−5.81	−4.87	−5.27
	STR11	−4.90	−1.00	−5.83	−4.92	−5.35
	STR12	−4.90	−0.81	−5.80	−4.88	−5.28
	STR13	−4.90	−0.80	−5.74	−4.80	−5.22
	STR14	−7.15	−1.45	−7.21	−6.33	−6.71
	STR15	−5.14	−0.89	−5.92	−4.95	−5.35
	STR16	−5.13	−0.82	−5.91	−4.98	−5.38
	STR17	−4.42	−0.73	−5.69	−5.01	−5.31
	STR18	−5.17	−0.79	−5.94	−4.99	−5.39

	STR19	−5.22	−0.80	−6.50	−5.67	−5.88
	STR20	−7.68	−1.92	−7.35	−6.58	−7.20
	STR21	−4.98	−0.90	−5.86	−4.92	−5.30
	STR22	−6.68	−0.90	−6.44	−6.30	−6.53
	STR23	−5.35	−0.95	−6.01	−5.13	−5.54
	STR24	−4.74	−0.76	−6.00	−5.82	−6.28
	STR25	−7.08	−2.74	−8.40	−7.67	−8.26
Ierissos Gulf	IER1	−5.74	−0.89	−6.04	−5.30	−5.70
	IER2	−4.44	**1.03***	−4.41	−4.62	−4.87
	IER3	−4.27	**1.24 ***	−4.08	−4.49	−4.79
	IER4	−4.49	0.07	−4.98	−5.46	−5.80
	IER5		−1.84	−7.70	−8.44	−10.37
	IER6	−2.61	**2.85 ****	−3.21	−4.03	−4.26
	IER7	−3.96	**1.66***	−3.79	−4.47	−4.62
	IER8	−2.41	**3.37 *****	−3.15	−5.10	−6.44
	IER9	−4.23	**1.19 ***	−4.24	−4.65	−5.03
	IER10	−7.04	−2.03	−7.17	−6.80	−7.59

Bolded values indicate site pollution according to the Müller’s scale (*moderate, ** moderate/strong, *** strong pollution).

Table S2. Potential risk factor (PRF$_i$) and potential ecological risk index (PERI) of heavy metals in sediment samples of three investigated gulfs, North Aegean Sea, Greece.

**Sampling location**	**Site**	**PRF$_i$**					**PERI**
		Cu	Pb	Zn	Cr	Ni	
Kavala Gulf	KAV1	0.07	1.20	0.02	0.02	0.01	1.31
	KAV2	0.01	0.63	0.01	0.01	0.00	0.66
	KAV3	0.05	1.20	0.02	0.02	0.01	1.29
	KAV4	0.03	1.13	0.01	0.02	0.01	1.19
	KAV5	0.01	0.92	0.01	0.01	0.00	0.95
	KAV6	0.04	1.28	0.01	0.02	0.01	1.36
	KAV7	0.01	0.87	0.01	0.01	0.00	0.90
	KAV8	0.19	2.90	0.02	0.03	0.01	3.15
	KAV9	0.16	2.72	0.02	0.02	0.00	2.92
	KAV10	0.08	1.46	0.01	0.04	0.02	1.60
	KAV11	0.19	1.34	0.02	0.04	0.02	1.60
	KAV12	0.30	1.95	0.02	0.03	0.01	2.31
	KAV13	1.06	7.06	0.19	0.04	0.01	8.35
	KAV14	0.27	1.11	0.02	0.08	0.05	1.53
	KAV15	0.16	1.11	0.02	0.06	0.04	1.39
	KAV16	0.04	0.96	0.01	0.03	0.01	1.06
	KAV17	0.11	1.40	0.02	0.05	0.03	1.60
	KAV18	0.26	1.66	0.02	0.06	0.04	2.04
	KAV19	0.15	2.01	0.02	0.05	0.04	2.28
	KAV20	0.19	2.78	0.03	0.06	0.05	3.10
	KAV21	0.22	2.27	0.03	0.06	0.04	2.63
	KAV22	0.20	2.23	0.03	0.07	0.05	2.56
	KAV23	0.14	1.62	0.02	0.06	0.04	1.89
	KAV24	0.18	2.21	0.03	0.06	0.05	2.53
	KAV25	0.20	1.81	0.02	0.09	0.05	2.18
Strymonikos Gulf	STR1	0.11	1.36	0.02	0.07	0.06	1.61
	STR2	0.07	0.81	0.01	0.05	0.04	0.98
	STR3	0.20	1.55	0.02	0.09	0.07	1.93
	STR4	0.29	2.58	0.02	0.09	0.08	3.07
	STR5	0.24	2.74	0.02	0.08	0.06	3.14
	STR6	0.25	3.21	0.02	0.09	0.07	3.65
	STR7	0.05	1.03	0.01	0.03	0.02	1.14
	STR8	0.19	2.86	0.02	0.07	0.06	3.21
	STR9	0.24	3.18	0.02	0.09	0.07	3.60
	STR10	0.25	3.70	0.03	0.10	0.08	4.16
	STR11	0.25	3.75	0.03	0.10	0.07	4.20
	STR12	0.25	4.28	0.03	0.10	0.08	4.73
	STR13	0.25	4.31	0.03	0.11	0.08	4.78
	STR14	0.05	2.75	0.01	0.04	0.03	2.88
	STR15	0.21	4.04	0.02	0.10	0.07	4.45
	STR16	0.21	4.26	0.03	0.09	0.07	4.66
	STR17	0.35	4.53	0.03	0.09	0.08	5.08
	STR18	0.21	4.33	0.02	0.09	0.07	4.73
	STR19	0.20	4.32	0.02	0.06	0.05	4.64
	STR20	0.04	1.99	0.01	0.03	0.02	2.08
	STR21	0.24	4.02	0.03	0.10	0.08	4.46
	STR22	0.07	4.02	0.02	0.04	0.03	4.18
	STR23	0.18	3.88	0.02	0.09	0.06	4.23
	STR24	0.28	4.44	0.02	0.05	0.04	4.83
	STR25	0.06	1.13	0.00	0.01	0.01	1.21
Ierissos Gulf	IER1	0.14	4.05	0.02	0.08	0.06	4.35
	IER2	0.35	15.29	0.07	0.12	0.10	15.93
	IER3	0.39	17.77	0.09	0.13	0.11	18.49
	IER4	0.33	7.86	0.05	0.07	0.05	8.37
	IER5	0.00	2.10	0.01	0.01	0.00	2.11
	IER6	1.23	54.10	0.16	0.18	0.16	55.83
	IER7	0.48	23.73	0.11	0.14	0.12	24.58
	IER8	1.41	77.54	0.17	0.09	0.03	79.24
	IER9	0.40	17.14	0.08	0.12	0.09	17.83
	IER10	0.06	1.84	0.01	0.03	0.02	1.95

Bolded values indicate site considerable risk.
